# Galectin-1 Induces the Production of Immune-Suppressive Cytokines in Human and Mouse T Cells

**DOI:** 10.3390/ijms252211948

**Published:** 2024-11-07

**Authors:** Kimberly D. Herman, Ian Holyer, Duncan C. Humphries, Anna Adamska, James A. Roper, Kristoffer Peterson, Fredrik R. Zetterberg, Anders Pedersen, Alison C. MacKinnon, Robert J. Slack

**Affiliations:** 1 Galecto Biotech AB, Cobis Science Park, Ole Maaloes Vej 3, DK-2200 Copenhagen, Denmarka.mackinnon6@icloud.com (A.C.M.); 2 Bioresearch and Veterinary Services, University of Edinburgh, The Chancellor’s Building, 49 Little France Cresent, Edinburgh EH16 4SB, UK

**Keywords:** galectin-1, immune-suppressive cytokines, T cells, cancer

## Abstract

Galectin-1 is implicated in several pro-tumourigenic mechanisms and is considered immune-suppressive. The pharmacological inhibition of galectin-1 may be beneficial in cancers in which galectin-1 is overexpressed and driving cancer progression. This study aimed to further characterise the immunosuppressive cytokines influenced by galectin-1 in in vitro immune cell cultures and an in vivo inflammatory model using a recently discovered selective inhibitor of galectin-1, GB1908. To enable a translational approach and link mouse and human pharmacology, anti-CD3/anti-CD28 stimulated T cells cultured from human whole blood and mouse spleens were compared. For in vivo studies of T cell-mediated inflammation, the concanavalin-A (Con-A) mouse model was used to induce a T lymphocyte-driven acute liver injury phenotype. The inhibition of galectin-1 with GB1908 reduced IL-17A, IFNγ and TNFα in a concentration-dependent manner in both mouse and human T cells in vitro. The immunosuppressive cytokines measured in Con-A-treated mice were all upregulated compared to naïve mice. Subsequently, mice treated with GB1908 demonstrated a significant reduction in IL-17A, IFNγ, IL-6 and TNFα compared to vehicle-treated mice. In conclusion, galectin-1 induced the production of several important immune-suppressive cytokines from T cells in vitro and in vivo. This result suggests that, in the context of cancer therapy, a selective galectin-1 could be a viable approach as a monotherapy, or in combination with chemotherapeutic agents and/or checkpoint inhibitors, to enhance the numbers and activity of cytotoxic T cells in the tumour microenvironment of high galectin-1 expressing cancers.

## 1. Introduction

Galectins are a family of lectins that mediate numerous biological functions in both health and disease. Due to their solubility, galectins are present in the extracellular space, cytosol and nucleus and can act both intra- and extracellularly. Galectins bind beta-galactoside sugars, a common glycan modification on many cell surface receptors, modulating cellular functions by altering receptor-mediated signal transduction pathways [1]. Galectins can also bind intracellular interactors in a carbohydrate-independent manner, with binding partners varying between different galectin family members [2]. The 15 galectin family members (11 in humans) are classified by the number and arrangement of their carbohydrate recognition domains (CRDs). Prototypical galectins have one and are found as monomers or homodimers (these include galectin-1 in addition to galectin-2, -5, -7, -10, -11, -13, -14 and -15); the chimeric galectin-3 has one CRD and an amino-terminal domain that can form oligomers; and the tandem repeat galectins (galectin-4, -6, -8, -9 and -12) contain two distinct CRDs connected by an amino acid linker and are present as monomers or dimers [1].

Galectin-1 regulates both physiological and disease-related processes, including tissue growth, cellular proliferation, apoptosis, vascularisation, inflammation and carcinogenesis [3]. In inflammation, galectin-1 is generally considered immunosuppressive due to its ability to induce apoptosis of effector T cells, antagonise T cell receptor signalling, block T cell-extracellular matrix interactions and activate regulatory T cell populations [4,5]. In a tumour microenvironment (TME), this suppression of T cells, in particular, may contribute to the persistence of tumours. Although some studies have begun to investigate the effects of galectin-1 on T cell cytokine production, these have been limited to in vitro and, in some cases, the use of exogenous lectin with non-specific inhibitors [6,7]. Therefore, in this study, we aimed to further characterise the immunosuppressive effects of galectin-1 on T cells by measuring key cytokines in vitro and in vivo following pharmacological intervention with a selective galectin-1 tool compound [8]. In addition, the translation between mouse and human T cells was also investigated to further aid future applications in a clinical setting.

## 2. Results and Discussion

### 2.1. Galectin-1 Inhibition Reduces Immune-Suppressive Cytokines in Both Mouse and Human Stimulated T Cells

**Figure 1:**
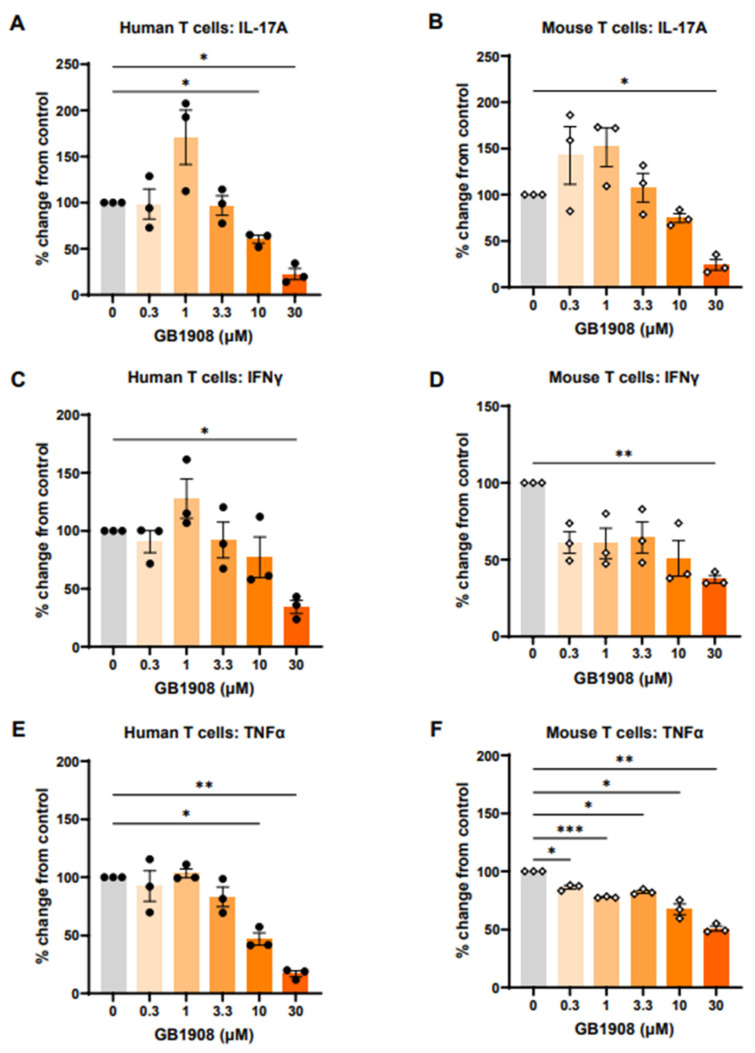
Galectin-1 inhibition reduces IL-17A, IFNγ and TNFα production in stimulated mouse and human T cells. T lymphocytes isolated from human whole blood (**A**,**C**,**E**) or mouse spleens (**B**,**D**,**F**) were stimulated with anti-CD3 and anti-CD28, along with a dose range of GB1908 (left column). IL-17A (**A**,**B**), IFNγ (**C**,**D**) and TNFα (**E**,**F**) measured in supernatants after 48 h. Bars show mean ± SEM, each dot representing one donor (n = 3). Data shown as % change from control (DMSO)-treated cells for each donor. Statistical analysis completed using repeated measures one-way ANOVA and Dunnett’s post-test (* *p* < 0.05, ** *p* < 0.01, *** *p* < 0.001).

To initially investigate the impact of galectin-1 on immunosuppressive cytokines produced from T cells, in vivo mouse models were considered. There are, however, gaps in the literature as to how closely mouse galectins function in the same way as human galectins. Therefore, prior to in vivo studies, in vitro investigations were used to determine whether galectin-1 inhibition results in comparable changes in cytokine production in mouse and human T cells. T cells cultured from human whole blood and mouse spleen were stimulated with anti-CD3 and anti-CD28 to induce cytokine production and were co-cultured with a range of GB1908 concentrations for 48 h. The inhibition of galectin-1 with GB1908 reduced IL-17 (Figure 1A,B), IFNγ (Figure 1C,D) and TNFα (Figure 1E,F) in a concentration-dependent manner in both mouse and human T cells, showing that this compound is a useful tool in both mouse and human experimental models.

### 2.2. Galectin-1 Inhibition Reduces Immune-Suppressive Cytokines in an Acute T Cell Driven Inflammatory Mouse Model

**Figure 2:**
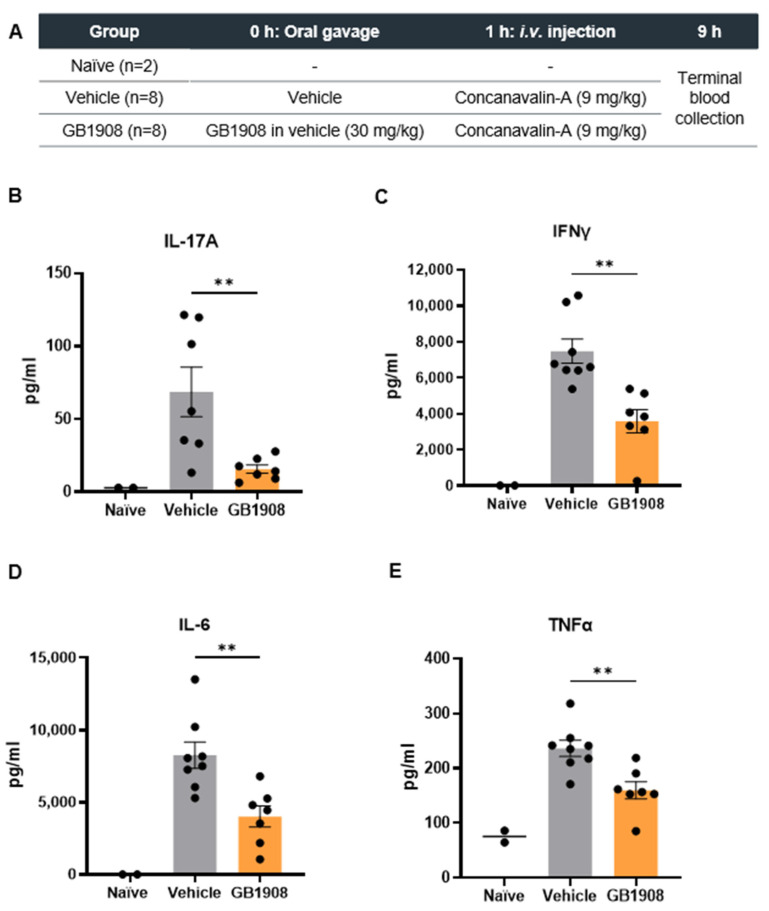
Galectin-1 inhibition reduces cytokine production in the mouse concanavalin-A model of acute inflammation. Summary of the Con-A study (**A**). IL-17A, IFNγ, IL-6 and TNFα were measured in terminal blood samples by BioLegend^®^ LEGENDPlex™ assay (**B**–**E**). Bars show mean ± SEM, and each dot represents one mouse. Statistical analysis completed using unpaired Student’s *t*-test comparing vehicle vs. GB1908 (** *p* < 0.01).

As GB1908 showed similar effects at inhibiting cytokines in activated T cells from both mouse and human models, an in vivo mouse model of T cell-mediated inflammation was utilised. Con-A induces acute liver injury by directly activating T lymphocytes, which are recruited to the liver [9]. Elevated inflammatory cytokines from the resulting inflammatory response can be measured in the circulation. The effects of galectin-1 inhibition on cytokines were assessed in this model by pre-treating mice orally with GB1908 or vehicle (control), one hour prior to Con-A injection (Figure 2A). Terminal blood samples were collected for analysis 8 h after Con-A injection. All cytokines measured were upregulated in Con-A treated mice in comparison to naïve mice that did not receive Con-A (Figure 2B–E). IL-17A, IFNγ, IL-6 and TNFα were all reduced in GB1908 treated mice in comparison to vehicle treated mice (Figure 2B–E). Although this model has been shown to be driven by T cell-mediated inflammation, there is potential that some of the galectin-1 effects on the measured cytokines could, in part, be contributed from other immune cell types.

In our investigations, several cytokines known to be immune-suppressive in the TME were reduced with a galectin-1 blockade. IL-17A is implicated in tumour progression in a wide range of cancer types through various mechanisms, including the upregulation of cancer cell migration and metastasis, the recruitment of myeloid-derived suppressor cells to induce angiogenesis and suppress antitumour immunity and the increase of PD-L1 expression [10,11,12]. Increased PD-L1 promoted resistance to immune checkpoint inhibitor therapy, and blocking IL-17A improved the efficacy of anti-PD-1 therapy in mouse models of colorectal cancer [13]. In murine myeloma transplant models, the pharmacological inhibition or genetic ablation of IL-17A improved outcomes [14]. In breast cancer, IL-17 expression by gamma-delta T cells resulted in the recruitment of a neutrophil population that supresses cytotoxic-T cells, promoting metastasis [15]. Similarly, IL-6 is also associated with poorer outcomes in many cancers. IL-6 drives the JAK/STAT3 signalling pathway, which suppresses tumour-killing NK cells, effector T cells and dendritic cells, whilst upregulating regulatory T cells and myeloid-derived suppressor cells to create an immunosuppressive environment [16]. High IL-6 also correlates with poor response to atezolizumab (anti-PD-L1 antibody) in advanced breast, kidney and bladder cancers in patients, while, in mouse models, a combined blockade of PD-L1 and IL-6 receptor improved outcomes compared to atezolizumab alone [17]. IL-6 production in tumour cells and associated fibroblasts, endothelial cells and dendritic cells was upregulated by IL-17, and, in murine models, an IL-6 blockade reduced Th17 cells and improved outcomes [18]. IL-6 levels were investigated in the previous in vitro T cell investigations but as expected, were below the level of detection, further strengthening the view that the contribution of this cytokine was via release from the additional cell types present in vivo.

For IFNγ and TNFα, the story is somewhat less straightforward in terms of the balance between the pro- and anti-tumour effects that these cytokines drive, and this phenomenon is likely dependent on the timing, concentration, and location of their production. IFNγ is considered a key player in the anti-tumour immune response, due to its ability to induce tumour cell apoptosis and senescence, polarise immune cells to a pro-inflammatory phenotype and inhibit angiogenesis [19]. There is evidence, however, that IFNγ regulates pro-tumourigenic mechanisms, with several studies attributing PD-L1 upregulation and immune escape directly due to IFNγ and cytotoxic T cells [20,21]. TNFα is also connected to both pro- and anti-tumour mechanisms. As the name suggests, TNFα acts directly on cancer cells to induce necrosis when presented at high concentrations. Pro-tumour mechanisms regulated by TNFα include promoting resistance to chemotherapeutic agents and checkpoint inhibitor therapy, increasing immune-suppressive phenotypes and promoting metastasis [22,23]. The significance of the suppression of IFNγ and TNFα by galectin-1 blockade and the role that they may play in the reduction in tumour growth is therefore still unclear and would require further investigation.

## 3. Materials and Methods

### 3.1. Human and Mouse T Cell Assays

Human and mouse T cell in vitro assays were carried out by Sygnature Discovery (Nottingham, UK). All assay reagents and plasticware were obtained from Thermo Fisher Scientific (Waltham, MA, USA) unless otherwise stated. For human assays, T cells were isolated from whole blood obtained from donors from the Sygnature Blood Donor panel, using the EasySep™ Human T cell Isolation Kit (STEMCELL Technologies UK Ltd., Cambridge, UK), as per manufacturer’s instructions. For mouse assays, T cells were isolated from the spleens of 6–12-week-old male BALB/c mice, sourced from Charles River UK, using the EasySep™ Mouse T cell Isolation Kit (STEMCELL Technologies UK Ltd., Cambridge, UK), as per manufacturer’s instructions. Isolated cells were resuspended in media (RPMI 1640 with a 10% inactivated foetal bovine serum, 1% penicillin–streptomycin, 1% GlutaMax and 55 µM beta-mercaptoethanol in mouse cell media only). The 96-well plates were coated with a 5 µg/mL anti-CD3 antibody (OKT3 clone). The selective galectin-1 inhibitor GB1908 [8] was synthesised by the Galecto Biotech AB Medicinal Chemistry Department, and stocks were prepared in 100% DMSO. GB1908 was diluted in media and added to wells at the desired concentrations with a final assay concentration of 0.5% DMSO. Cells were added to wells at a concentration of 100,000 cells/well and incubated for 30 min (37 °C, 5% CO_2_) before the anti-CD28 antibody (CD28.2 clone) was added to a final concentration of 1 µg/mL. Cells were incubated for a further 48 h. Then, supernatants were harvested and stored at −80 °C until analysis. Human and mouse 5-plex U-Plex kits for the analysis of tumour necrosis factor-α (TNFα), interleukin-6 (IL-6), interleukin-17A (IL-17A) and interferon-γ (IFNγ) were obtained from Meso Scale Discovery (Rockville, MD, USA) for cytokine analysis as per manufacturer’s instructions, and plates were read using the MESO QuickPlex SQ120MM instrument (Meso Scale Discovery, Rockville, MD, USA). Data were analysed using Meso Scale Discovery Workbench 4 software.

### 3.2. Concanavalin-A Mouse Models

Mouse concanavalin-A (Con-A) models were carried out at the University of Edinburgh, under a project licence approved by the local Animal Welfare and Ethical Review body and issued in accordance with the Animals (Scientific Procedures) Act 1986. Male C57BL/6 mice (purchased from Charles River, Harlow UK) received GB1908 (30 mg/kg) dissolved in vehicle (84% PEG + 15% Solutol HS15 + 1% Tween-20) or vehicle only via oral gavage at a concentration of 10 mL/kg. After 1 h, mice received Con-A (Sigma, St. Louis, MO, USA), C5275; 9 mg/kg, dissolved at a concentration of 1.5 mg/mL in sterile saline) i.v. via the peripheral vein. Mice were monitored throughout this study and were euthanised prior to the end of this study if moderate clinical signs were breached. At 8 h post-Con-A injection, mice were culled by anaesthetic overdose (i.p.), and blood was collected from the vena cava. Blood samples were centrifuged at 400× *g* with no brake for 10 min, and the plasma was removed and stored at −20 °C until analysis. Plasma cytokines (TNFα, IL-6, IL-17A and IFNγ) were measured using a BioLegend^®^ LEGENDPlex™ assay (Mouse Inflammation Panel) (San Diego, CA, USA), as per the manufacturer’s protocols. Data were acquired using a BD LSR Fortessa (5 laser) analyser (BD Biosciences, Franklin Lakes, NJ, USA). After acquisition, data were analysed using BioLegend^®^ Qognit software version 1 (San Diego, CA, USA).

### 3.3. Data Analysis

All data were analysed using GraphPad Prism 10.2 (GraphPad Software, San Diego, CA, USA) and were expressed as mean ± SEM. Statistical analyses were completed using a Student’s *t*-test for comparing two datasets and a one-way analysis of variance (ANOVA) for the comparison of more than two datasets, with an appropriate post-test completed where significance was observed.

## 4. Conclusions

Our research demonstrates that, via inhibition with the selective galectin-1 inhibitor GB1908, galectin-1 induces the production of several important immune-suppressive cytokines from T cells in vitro and in vivo. The current evidence may suggest that a galectin-1 inhibitor in combination with existing therapies, such as chemotherapeutic agents and/or checkpoint inhibitors, or even as a monotherapy, may reduce the pro-tumour effects of IL-17A, IL-6, IFNγ and TNFα and turn a “cold”, immunosuppressed TME into a “hot”, inflamed, cytotoxic T cell-rich TME, resulting in increased tumour cell killing and enhanced cancer prognosis and survival.

## Data Availability

Data is contained within the article and supplementary material.

## Supplementary Materials

The supporting information can be downloaded at:https://www.mdpi.com/article/10.3390/ijms252211948/s1.
