# Supplementary material for: Galectin-1 Induces the Production of Immune-Suppressive Cytokines in Human and Mouse T Cells
Source: Int J Mol Sci. 2024 Nov 7;25(22):11948. doi: 10.3390/ijms252211948 (PMC11593614; doi:10.3390/ijms252211948)
Supplement: Supplementary file 1 [file ijms-25-11948-s001.zip › ijms-3246277-supplementary.pdf]

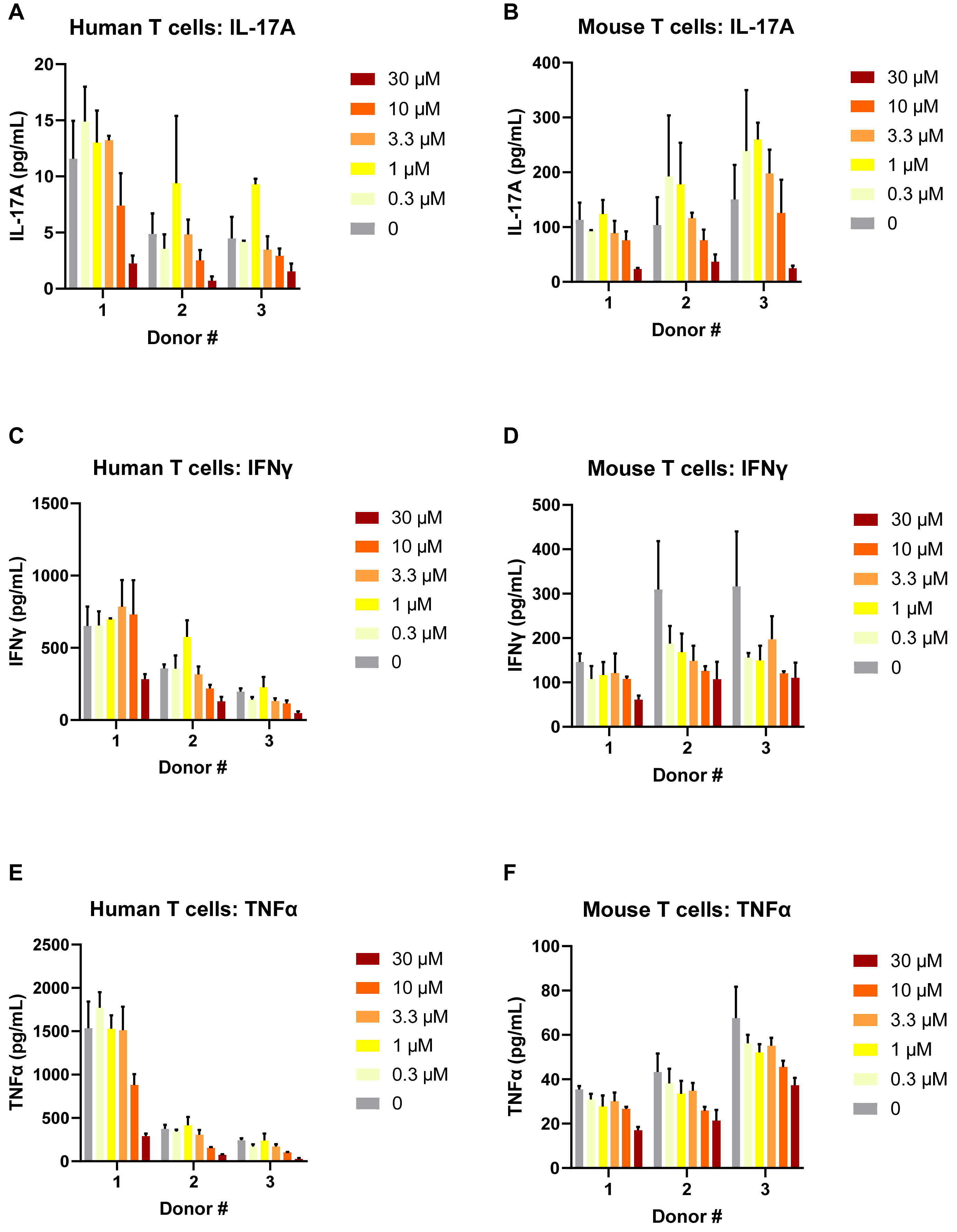

Supplementary Figure S1. Raw data for IL-17A, IFNγ and TNFα production in stimulated mouse and human T cells across donors following GB1908 treatment. T lymphocytes isolated from human whole blood (A, C, E) or mouse spleen (B, D, F) were stimulated with anti-CD3 and anti-CD28, along with a dose range of GB1908. IL-17A (A-B), IFNγ (C-D) and TNFα (E-F) measured in supernatants after 48 hours. Bars show mean ± SD with n=3 per donor.
